# Immune-Related Comorbidities in Childhood-Onset Obsessive Compulsive Disorder: Lifetime Prevalence in the Obsessive Compulsive Disorder Collaborative Genetics Association Study

**DOI:** 10.1089/cap.2018.0140

**Published:** 2019-10-07

**Authors:** Clara Westwell-Roper, Kyle A. Williams, Jack Samuels, O. Joseph Bienvenu, Bernadette Cullen, Fernando S. Goes, Marco A. Grados, Daniel Geller, Benjamin D. Greenberg, James A. Knowles, Janice Krasnow, Nicole C. McLaughlin, Paul Nestadt, Yin-Yao Shugart, Gerald Nestadt, S. Evelyn Stewart

**Affiliations:** ^1^ Department of Psychiatry, Faculty of Medicine, British Columbia Children's Hospital, University of British Columbia, Vancouver, Canada.; ^2^ Department of Psychiatry, Harvard Medical School, Massachusetts General Hospital, Boston, Massachusetts.; ^3^ Department of Psychiatry and Behavioral Sciences, Johns Hopkins University School of Medicine, Baltimore, Maryland.; ^4^ Department of Psychiatry and Human Behavior, Brown Medical School, Butler Hospital, Providence, Rhode Island.; ^5^ Department of Psychiatry, University of Southern California School of Medicine, Los Angeles, California.; ^6^ Unit of Statistical Genomics, Division of Intramural Research, National Institute of Mental Health, Bethesda, Maryland.

**Keywords:** obsessive compulsive disorder, childhood-onset, comorbidity, immune system diseases, autoimmune diseases, communicable diseases, inflammation

## Abstract

***Objective:*** To evaluate the lifetime prevalence of infectious, inflammatory, and autoimmune disorders in a multisite study of probands with childhood-onset obsessive compulsive disorder (OCD) and their first-degree relatives.

***Methods:*** Medical questionnaires were completed by 1401 probands and 1045 first-degree relatives in the OCD Collaborative Genetics Association Study. Lifetime prevalence of immune-related diseases was compared with the highest available population estimate and reported as a point estimate with 95% adjusted Wald interval. Worst-episode OCD severity and symptom dimensions were assessed with the Yale–Brown Obsessive Compulsive Scale (YBOCS) and Symptom Checklist (YBOCS-CL).

***Results:*** Probands reported higher-than-expected prevalence of scarlet fever (4.0 [3.1–5.2]% vs. 1.0%–2.0%, *z* = 1.491, *p* < 0.001, *n* = 1389), encephalitis or meningitis (1.4 [0.9–2.1]% vs. 0.1%–0.4%, *z* = 5.913, *p* < 0.001, *n* = 1393), rheumatoid arthritis (1.1 [0.6–2.0]% vs. 0.2%–0.4%, *z* = 3.416, *p* < 0.001, *n* = 949) and rheumatic fever (0.6 [0.3–1.2]% vs. 0.1%–0.2%, *z* = 3.338, *p* < 0.001, *n* = 1390), but not systemic lupus erythematosus, diabetes, asthma, multiple sclerosis, psoriasis, or inflammatory bowel disease. First-degree relatives reported similarly elevated rates of scarlet fever, rheumatic fever, and encephalitis or meningitis independent of OCD status. There was no association between worst-episode severity and immune-related comorbidities, although probands reporting frequent ear or throat infections had increased severity of cleaning-/contamination-related symptoms (mean factor score 2.5 ± 0.9 vs. 2.3 ± 1.0, *t* = 3.183, *p* = 0.002, *n* = 822).

***Conclusion:*** These data suggest high rates of streptococcal-related and other immune-mediated diseases in patients with childhood-onset OCD and are consistent with epidemiological studies in adults noting familial clustering. Limitations include potential reporting bias and absence of a control group, underscoring the need for further prospective studies characterizing medical and psychiatric disease clusters and their interactions in children. Such studies may ultimately improve our understanding of OCD pathogenesis and aid in the development of adjunctive immune-modulating therapeutic strategies.

## Introduction

Obsessive compulsive disorder (OCD) affects 1%–3% of children worldwide and causes significant distress for patients and families (Hollander et al. 2016). Despite effective cognitive behavioral therapy and pharmacotherapy, many patients experience a chronic, episodic course with long-term functional impairment (Stewart et al. 2004; Mancebo et al. 2014).

While the underlying pathophysiological mechanisms remain to be elucidated, meta-analyses of family studies suggest heritability may be as high as 50%–60% in childhood-onset OCD (Nestadt et al. 2000; Mataix-Cols et al. 2013). Genetic association studies have implicated single nucleotide polymorphisms in genes involved in glutaminergic transmission as well as immune function (den Braber et al. 2016), although none has yet reached genome-wide significance (Stewart et al. 2013; Taylor 2013; Mattheisen et al. 2015; International Obsessive Compulsive Disorder Foundation Genetics Collaborative [IOCDF-GC] and OCD Collaborative Genetics Association Studies [OCGAS] 2018).

Increasing evidence suggests a potential role for the immune system in OCD pathogenesis. Children with sudden onset of OCD and/or motor tics and evidence of recent streptococcal infection may meet criteria for pediatric autoimmune neuropsychiatric disorder associated with streptococcal infections (PANDAS) (Williams and Swedo 2015). Early studies suggested that this subset of patients may develop postinfectious immune-mediated damage to the basal ganglia, as described for Syndenham's chorea, a neuropsychiatric complication of rheumatic fever (RF) (Williams and Swedo 2015).

A potential role for infection as a trigger for neuropsychiatric symptoms is supported by epidemiological evidence of an association between throat infections and mental disorders, in particular OCD (Orlovska et al. 2017). Although controversial, definitive evidence for causative pathogenic autoantibodies in humans is lacking (Dale et al. 2005; Morer et al. 2008) and PANDAS—together with pediatric acute-onset neuropsychiatric syndrome (PANS)—may account for as few as 5% of children attending some general pediatric OCD outpatient clinics (Jaspers-Fayer et al. 2017).

Data from studies in patients with “classic” OCD also suggest an association with aberrant innate immune activation. This includes altered levels of systemic cytokines, such as tumor necrosis factor-α and interlelukin-1β (Gray and Bloch 2012; Mitchell and Goldstein 2014; Rao et al. 2015; Simsek et al. 2016) as well as lower-than-expected immunoglobulin titers (Kawikova et al. 2010; Williams 2016; Calaprice et al. 2017), including immunoglobulin A (IgA), which provides protection at mucosal surfaces and can be produced through T cell-independent mechanisms (Macpherson et al. 2011). Moreover, a recent positron emission tomography imaging study in adults with OCD—many with childhood-onset—showed increased volume of translocator protein-18 (TSPO) distribution in cortico-striato-thalamo-cortical circuits, implicating widespread microglial activation (Attwells et al. 2017). Further support for a potential causative or perpetuating role of inflammation due to abnormal innate immune cell activity was provided by a double-blind, placebo-controlled randomized controlled trial of 50 adult outpatients with moderate-to-severe OCD that found modest symptom improvement with the nonsteroidal anti-inflammatory drug (NSAID) celecoxib as an adjuvant to fluvoxamine (Shalbafan et al. 2015).

Patients with autoimmune disorders such as systemic lupus erythematosus (SLE) and RF—and in some cases, their first-degree relatives—have higher rates of comorbid OCD and anxiety disorders compared with the general population (Slattery et al. 2004; Hounie et al. 2007). A recent large birth cohort study based on Swedish National Register data also suggested increased rates of multiple autoimmune diseases among adults with OCD as well as their first-degree relatives, including inflammatory bowel disease (IBD), Hashimoto's thyroiditis, celiac disease, psoriasis, type 1 diabetes, idiopathic thrombocytopenic purpura, Sjögren's syndrome, and Guillan-Barré syndrome in addition to scarlet fever (Mataix-Cols et al. 2017).

Potential mechanisms leading to familial associations between immune-mediated diseases and OCD include dual genetic and environmental susceptibility and exposure to maternal antibodies (Vincent et al. 2003) or plasma cytokines (Golan et al. 2005) leading to altered perinatal brain development. While the current PANS consensus guidelines recommend clinicians take a thorough personal and family history of autoimmune and infectious diseases in children with OCD (Chang et al. 2015), little is known about the prevalence of these conditions in this population (Perez-Vigil et al. 2016).

Previous studies have implicated frequent infections, low immunoglobulin titers, and higher rates of atopy among OCD probands together with increased prevalence of autoimmune diseases among their mothers (Murphy et al. 2010; Stagi et al. 2014; Yuce et al. 2014; Frankovich et al. 2015; Calaprice et al. 2017) (Supplementary Table S1). However, these reports are limited by the absence of control or population data, measurement of hematologic parameters only in the setting of acute symptom flares, restriction to PANS or PANDAS subtypes rather than heterogeneous or adult OCD samples, or small sample sizes. This study examined the prevalence of self-reported immune-mediated illnesses in patients with childhood-onset OCD not selected for PANDAS or PANS and compared these with expected ranges derived from a review of published population estimates. We hypothesized that shared genetic and environmental predisposing factors may contribute to increased rates of immune-related diseases among heterogeneous patients with childhood-onset OCD.

## Methods

### Study cohort

The multisite OCGAS comprised patients with OCD onset before the age of 18, recruited between 2007 and 2012 (Mattheisen et al. 2015). Study design and methods have been described previously (Samuels et al. 2006). A total of 1065 families were included; of these, 621 families were recruited and assessed specifically for this study at one of the five participating recruitment sites or the National Institute of Mental Health (NIMH), whereas 444 families had been evaluated previously in an earlier study at one of the collaborating sites. The complete sample comprised 460 trios (an affected proband and both parents), 155 pedigrees with a proband and an unaffected sibling, 450 families with another structure, and an additional 192 singleton probands.

Inclusion criteria were as follows: (1) Diagnostic and Statistical Manual of Mental Disorders, 4th ed (DSM-IV, American Psychiatric Association 1994) OCD diagnosis as established by standardized interview by a psychiatrist or clinical psychologist using the Structured Clinical Interview for DSM-IV with additional symptom and diagnostic information as previously described (Mattheisen et al. 2015), and (2) onset of obsessions and/or compulsions before age 18.

Exclusion criteria were as follows: (1) subjects with schizophrenia, severe intellectual disability, or another condition preventing evaluation; (2) Tourette disorder without OCD; (3) OCD-like symptoms occurring exclusively in the context of depression; and (4) previous diagnosis of brain pathology, including brain tumors, Huntington's disease, Parkinson's disease, or Alzheimer's disease. Final diagnostic status was assigned based on the consensus of two psychiatrists or psychologists reviewing the case independently followed by review by one of five consensus psychiatrists as described previously (Samuels et al. 2006). Probands with a diagnosis of “probable” or “definite” OCD were included.

Study protocols were approved by the Institutional Review Boards at all participating sites. All participants provided written informed consent.

### Study measures and instruments

OCD assessment was performed as described previously and adapted from the Schedule for Affective Disorders and Schizophrenia-Lifetime Version (SADS-LA-R) (Samuels et al. 2006). The Structured Clinical Interview for DSM-IV Axis I Disorders (SCID-I) is a semistructured interview for making major DSM-IV Axis I diagnoses and was used in assessment of psychiatric comorbidities (First et al. 2002).

OCD severity for the worst episode was assessed using the Yale–Brown Obsessive Compulsive Scale (YBOCS) (Goodman et al. 1989). Severity ratings for specific symptom dimensions were determined as described previously using the YBOCS Symptom Checklist (YBOCS-CL) to score each of four factors: forbidden thoughts (aggressive, religious, and sexual obsession categories), symmetry (symmetry obsessions, checking, counting, ordering, and repeating compulsion categories), cleaning (contamination, somatic obsessions, and cleaning compulsion categories), and hoarding (hoarding obsession/compulsion categories) (Bloch et al. 2008).

Medical comorbidities were assessed by self report using a standard medical questionnaire (Supplementary Data). Selected chronic comorbidities specified a requirement for physician diagnosis, whereas acute or episodic conditions did not. Questionnaires assessing personal and family history of selected infectious, autoimmune, and inflammatory conditions were completed by probands and a subset of the questionnaire was also completed by first-degree relatives (Supplementary Data). These included (1) self-reported history of encephalitis or meningitis, frequent ear/throat infections (frequency not defined), scarlet fever, RF, and rheumatic heart disease in both probands and first-degree relatives; and (2) self-reported physician diagnosis of rheumatoid arthritis (RA), SLE, multiple sclerosis (MS), diabetes, asthma, psoriasis, ulcerative colitis, Crohn's disease, and Sydenham's chorea in probands only. No additional follow-up or verification of reported comorbidities was performed.

### Estimation of population prevalence

To determine expected population prevalence of the immune-related comorbidities included in the OCGAS medical questionnaire, a review of United States data was carried out using PubMed and Medline databases and annual reports published by the Centers for Disease Control and Prevention. Keywords and subject headings were used to identify studies of prevalence or incidence. Both population-based cross-sectional and cohort studies were included, with data based on self-report surveys, where available. When national data were unavailable, North American data were used. Broad ranges were used to include all frequencies and confidence intervals described in identified reports. Where incidence only was available, prevalence was determined by multiplying by the cohort's mean age, 27.8 years. These conservative lifetime prevalence values derived from incidence are therefore likely to be overestimates. Where indicated in Supplementary Table S2, under- or nonreporting of diseases limited the utility of available data.

### Data analyses

Statistical analyses were conducted using SPSS (Version 24, IBM Corporation). Prevalence was reported as an exact point estimate and 95% adjusted Wald interval (Agresti and Coull 1998). The valid percent was reported if data were missing, determined by excluding cases with either no response or with responses reported as “unsure.” Differences between means for continuous variables were analyzed with a two-sided student's *t*-test or Welch's *t*-test (Welch 1947) as indicated by the results of Levene's test for equal variances (Levene 1960). One-sample *z*-tests with binomial *p*-values were used to compare prevalence estimates with the maximum population estimate based on literature review. Given low frequencies for some conditions, differences among dichotomous categorical variables were analyzed with a two-sided Fisher's exact test. Pearson's chi-squared test was used to compare frequencies for nominal variables with two or more categories. Correction for multiple comparisons was applied as indicated using the Bonferonni correction with α = 0.05 for the indicated number (*n*) of tests (Benjamini and Hochberg 1995). Continuous variables are reported as mean ± standard deviation with 95% confidence intervals in square brackets.

## Results

### Participant demographics

Demographic information is shown in Table 1. A total of 1401 probands met inclusion criteria. Probands had an average age of 27.8 ± 14.1 years (median 25 years) with a history of symptom onset at 7.7 ± 3.5 years and diagnosis at 10.1 ± 3.7 years. One-third (33%) were age 18 or under at the time of assessment. Approximately half (56%) were female and 91% identified as Caucasian or White. At the time of assessment, females were on average older than males [mean age 29.7 ± 14.3 vs. 25.4 ± 13.4 years, *t*(1399) = −5.741, *p* < 0.001, *n* = 1401] and had experienced OCD symptom onset at a younger age [7.5 ± 3.3 vs. 8.1 ± 3.7 years, *t*(1239) = 3.369, *p* = 0.001, *n* = 1398].

**Table 1.** Demographic Information of Obsessive Compulsive Disorder Collaborative Genetics Association Study Participants

*Characteristic*	*Value*	n
Age of proband (years)		1401
Mean (SD)	27.8 (14.0)	
Range	6–82	
Gender (% of total)^a^		1401
Male	43.6	
Female	56.4	
Race/ethnicity (% of total)^b^		1394
Caucasian	91.5	
Other ^b^	8.5	
Age of OCD onset (years)^c^		1398
Mean (SD)	7.7 (3.5)	
Range	1–18	
YBOCS score		1248
Mean (SD)	29.1 (6.3)	
Range	3–40	
Psychiatric comorbidity (% of total)		
Tic disorder^d^	22.4	691
Mood disorder	64.9	1374
Anxiety disorder	66.7	1361
ADHD	15.3	1245
Eating disorder	8.4	1375
Psychotic disorder	0.4	1376

^a^ Percentages represent frequencies relative to *n* total respondents for each question.

^b^ Response choices included a mixture of race and ethnicity. Other ethnicities included Hispanic/Latin American (2.9%) and African American (1.5%).

^c^ Age of onset is based on age at initial symptom presentation. Age at which study participants first met DSM-IV diagnostic criteria for OCD was 10.1 (3.7) years (range 1–18 years, *n* = 1388).

^d^ A subset of patients was not assessed for all tic disorders and has therefore been omitted from the denominator.

YBOCS, Yale–Brown Obsessive Compulsive Scale; SD, standard deviation; OCD, obsessive compulsive disorder; ADHD, attention-deficit/hyperactivity disorder.

Mean YBOCS score for the worst episode was 29 ± 6.3, consistent with previous reports (Chen et al. 2017). Most participants (81%) had worst-episode YBOCS scores in the severe-to-extreme range of 24 and above. Two participants had scores in the subclinical range (0–7); omission of these individuals did not affect our reported results.

### Lifetime prevalence of immune-related diseases

Self-reported lifetime prevalence of immune-related comorbidities included in the medical questionnaire is shown in Table 2. There were no cases of rheumatic heart disease. The prevalence of Sydenham's chorea and MS (one and two cases, respectively) was not significantly different from zero. Lifetime prevalence of SLE, type 1 diabetes (based on insulin dependence under age 40), asthma, psoriasis, and IBD was within the range expected based on population data (reviewed in Supplementary Table S2). Higher-than-expected prevalence was reported for encephalitis or meningitis (1.4 [0.9–2.1]% vs. 0.1%–0.4%, *z* = 5.913, *p* < 0.001, *n* = 1393), scarlet fever (4.0 [3.1–5.2]% vs. 1.0%–2.0%, *z* = 1.491, *p* < 0.001, *n* = 1389), RF (0.6 [0.3–1.2]% vs. 0.1%–0.2%, *z* = 3.338, *p* < 0.001, *n* = 1390), and RA (1.1 [0.6–2.0]% vs. 0.2%–0.4%, *z* = 3.416, *p* < 0.001, *n* = 949).

**Table 2.** Lifetime Prevalence of Infectious and Immune-Mediated Diseases Among Obsessive Compulsive Disorder Collaborative Genetics Association Study Probands

	*Prevalence*^a^				*Comparison*	
*Medical comorbidity*	*Cases*	*% (CI)*^b^	n	*Population estimate (%)*	z	P
Infectious						
Encephalitis or meningitis	19	1.4 (0.9–2.2)^c^	1379	0.1–0.4	5.883	<0.001
Frequent ear/throat infections	495	35.9 (33.4–38.4)	1381	ND	NA	NA
Scarlet fever	55	4.0 (3.1–5.2)^c^	1375	1.0–2.0	5.297	<0.001
Autoimmune						
Rheumatic fever	8	0.6 (0.3–1.2)^c^	1376	0.1–0.2	3.321	0.009
Rheumatoid arthritis	10	1.1 (0.6–2.0)^c^	949	0.2–0.4	3.416	0.006
SLE	3	0.3 (0.1–1.0)	949	0.2–0.3	0.000	1.000
Type 1 diabetes^d^	5	0.5 (0.2–1.3)	951	0.2–0.6	0.399	0.690
Atopic						
Asthma	164	17.2 (14.9–19.7)	953	11.9–14.3	2.557	0.011
Inflammatory						
Psoriasis	21	2.2 (1.4–3.4)	951	2.6–3.7	0.775	0.438
Ulcerative colitis	6	0.6 (0.3–1.4)	949	0.4–0.8	0.692	0.489
Crohn's disease	5	0.5 (0.2–1.3)	947	0.4–0.8	0.488	0.626
IBD	10	1.1 (0.6–2.0)	945	0.8–1.3	0.543	0.587

Conditions with 95% CI containing 0 were not included in this table: Sydenham's chorea (0.1 [0.0–0.5]%, *n* = 1381) and multiple sclerosis (0.2 [0.0–0.8]%, *n* = 951). There were no reported cases of rheumatic heart disease.

^a^ Prevalence determined based on *n* = total number of respondents (denominator). There were no significant differences in prevalence based on gender when adjusted for multiple comparisons (α = 0.05, *n* = 12), although all eight cases of rheumatic fever were in females. Gender-specific prevalence estimates were 1.0 [0.5–2.1]% (*n* = 780) for females and 0 [0.0–0.5]% (*n* = 610) for males (*p* = 0.011, Fisher's exact test).

^b^ CI, 95% binomial confidence interval calculated by adjusted Wald formula.

^c^ Estimated prevalence greater than expected based on published population data. Note limited available data on scarlet fever incidence in the United States of America; expected lifetime prevalence at age 30 was estimated assuming highest recent international rates (Supplementary Table S2).

^d^ Estimate based on age of diabetes diagnosis at ≤40 years and insulin dependence.

ND, not determined; NA, not applicable; IBD, inflammatory bowel disease; SLE, systemic lupus erythematosus.

All cases of RF (*n* = 8/1390) were in females, yielding gender-specific prevalence estimates of 1.0 [0.5–2.1]% (*n* = 780) for females and 0.0 [0.0–0.5]% (*n* = 610) for males (*p* = 0.011, Fisher's exact test, not significant when corrected for multiple comparisons among all conditions in Table 2). Participants with OCD onset at 8 years or younger had a higher prevalence of tic disorders compared with those with later onset (30.0 [24.5–36.1]% vs. 18.7 [15.3–22.5]%, *n* = 687, *p* = 0.001 by Fisher's exact test), but no difference in prevalence of any immune-related comorbidity. Similarly, there was no significant effect of gender, ethnicity (Caucasian vs. other), or comorbid tic disorder on prevalence of encephalitis or meningitis, frequent ear/throat infections, scarlet fever, RF, RA, asthma, psoriasis, IBD, or the presence of at least one comorbidity.

### Immune-related disease prevalence in first-degree relatives

A subset of first-degree relatives also completed a portion of the medical questionnaire (*n* = 1045). Approximately 60% had probable or definite OCD, and these first-degree relatives were on average younger at the time of assessment than those without OCD [34.9 ± 19.2 vs. 50.3 ± 17.7 years, *t*(1043) = −13.974, *p* < 0.001, *n* = 1045]. Table 3 compares prevalence of immune-related comorbidities in first-degree relatives with and without OCD for conditions with available data from at least 5% of participants in each group. Similar to OCD-affected probands, the self-reported lifetime prevalence of RF, encephalitis or meningitis, and scarlet fever was greater than expected compared with population rates. There was no difference in prevalence between OCD-affected and unaffected first-degree relatives (Table 3), although the study was insufficiently powered to detect differences in prevalence less than two-fold for each condition.

**Table 3.** Lifetime Prevalence of Immune-Mediated Comorbidities Among First-Degree Relatives of Obsessive Compulsive Disorder Collaborative Genetics Association Study Probands With and Without Obsessive Compulsive Disorder

	*Prevalence in relatives with OCD*^a^			*Prevalence in relatives without OCD*			*Comparison*	
*Comorbidity*	*Cases*	*% (CI)*^b^	n	*Cases*	*% (CI)*^b^	n	*χ*^2^	P
Rheumatic fever	8	1.3 (0.6–2.6)^c^	607	9	2.2 (1.1–4.2)^b^	404	1.203	0.273
Rheumatic heart disease	4	0.7 (0.2–1.7)	609	2	0.5 (0.0–1.9)	405	0.002	0.968
Encephalitis or meningitis	9	1.5 (0.7–2.8)^c^	609	5	1.2 (0.4–3.0)^b^	404	0.002	0.968
Sydenham's chorea	1	0.2 (0.0–1.0)	611	0	0 (0.0–0.8)	404	0.028	0.867
Frequent ear or throat infections	168	27.9 (24.4–31.6)	603	89	22.4 (18.6–26.8)	397	0.003	0.958
Scarlet fever	23	3.8 (2.5–5.7)^c^	603	23	5.8 (3.9–8.6)^b^	397	0.021	0.885

^a^ Cases defined by definite or probable OCD. Prevalence determined based on *n* = total number of respondents (denominator).

^b^ CI, 95% binomial confidence interval calculated by adjusted Wald formula.

^c^ Prevalence greater than expected based on published data (Supplementary Table S2).

OCD, obsessive compulsive disorder.

Of the 413 mothers of probands who completed medical questionnaires, frequencies of RF, scarlet fever, frequent ear/throat infections, and encephalitis/meningitis were similar to those in the overall first-degree relative group. Very few mothers completed the second section of the questionnaire regarding physician-diagnosed autoimmune and inflammatory diseases; therefore, we could not estimate prevalence of maternal SLE, MS, RA, type 1 diabetes, or psoriasis.

### Association of immune-related diseases and OCD symptoms

Neither age at the time of diagnosis nor YBOCS score was related to the presence of individual comorbidities or their general categories (infectious, autoimmune/inflammatory, or any immune-related comorbidity) (Supplementary Table S3). We next determined OCD symptom scores based on a previous factor analysis of symptoms in the YBOCS-CL (Bloch et al. 2008). Participants reporting at least one immune-related comorbidity had slightly higher scores for forbidden thoughts, cleaning, and hoarding (Supplementary Table S3). These differences were primarily driven by frequent ear or throat infections, the only condition with an increased score for cleaning-related symptoms that remained significant after adjustment for multiple comparisons [2.5 ± 0.9 vs. 2.3 ± 1.0, *t*(644) = 3.183, *p* = 0.002, *n* = 822]. There was no difference in YBOCS score or symptom severity based on gender.

### Groupings of immune-related comorbidities

**FIG. 1.**
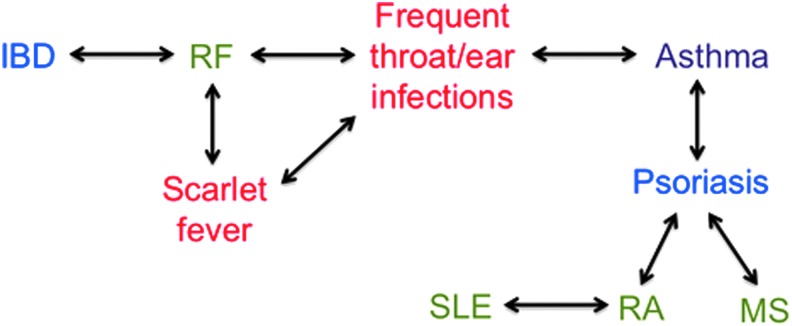
Associations among immune-related comorbidities in OCD Collaborative Genetics Association Study probands with childhood-onset OCD. Significant associations described in Supplementary Table S4 are depicted by two-way arrows. Comorbidities include diseases with autoimmune (green; SLE, RA, MS), infectious (red; ear/throat infections, scarlet fever), combined autoimmune and infectious (RF), atopic (purple; asthma), and inflammatory (blue; psoriasis, IBD) etiologies, although recent data suggest significant overlap in pathophysiology and genetic predisposition among these conditions. All except for an association between IBD and rheumatic fever have been reported for the general population. IBD, inflammatory bowel disease; SLE, systemic lupus erythematosus; RA, rheumatoid arthritis; MS, multiple sclerosis; RF, rheumatic fever; OCD, obsessive compulsive disorder.

To determine whether specific immune-related comorbidities tend to group together, we assessed the relative risk of each comorbidity given the presence of another using a pairwise comparison matrix for all conditions in Table 3. Frequent throat/ear infections, scarlet fever, and RF—all potential consequences of streptococcal infection—showed significant pairwise associations (Supplementary Table S4 and Fig. 1). Moreover, asthma was associated with frequent throat/ear infections; SLE with RA; psoriasis with RA, MS, and asthma; and RF with IBD. These data identify groups of conditions with potential shared etiology and common environmental and genetic risk factors.

## Discussion

This descriptive study used self-report medical questionnaires from a large cohort of patients with childhood-onset OCD to estimate the lifetime prevalence of immune-related medical comorbidities. Self-reported lifetime prevalence of encephalitis or meningitis, scarlet fever, RF, and RA was greater than expected in probands with OCD and in their OCD-affected and unaffected first-degree relatives. Similar rates of RF, encephalitis or meningitis, and scarlet fever were identified in first-degree relatives independent of OCD status, suggesting that—if reflective of true increased prevalence—such comorbidities may represent common familial traits and genetic vulnerability rather than state markers of disease.

Given the potential for reporting bias and lack of a control group in the present study, a replication sample would aid in providing further support for these findings. Nevertheless, these data constitute the largest descriptive study of selected immune-related diseases in childhood-onset OCD and point to a need for further controlled clinical studies to understand the potential interactions between OCD and comorbid medical conditions. To date, clinical evidence addressing comorbid autoimmune and inflammatory diseases in patients and family members with childhood-onset OCD has largely been limited to small studies focused on the putative PANS/PANDAS subtype (Supplementary Table S1).

Potential mechanisms for the association among immune-related comorbidities and OCD include shared genetic and environmental susceptibility, given associations between autoimmune disease in adults with OCD and their first-degree relatives (Mataix-Cols et al. 2017); maternal antibody transfer during pregnancy given increased frequency among mothers with autoimmune disorders compared with other first-degree relatives (Vincent et al. 2003), although this appears more relevant in tic disorders (Mataix-Cols et al. 2017); or exposure to dysregulated plasma cytokines (Golan et al. 2005) leading to altered brain development. For example, previous work has also suggested a familial relationship between obsessive compulsive spectrum disorders and RF (Seixas et al. 2008), an autoimmune disease thought to arise from humoral and cellular autoreactivity following group A streptococcal (GAS) pharyngitis (Bright et al. 2016).

While the heritability of acute RF has been estimated at 60%, few replicable genetic susceptibility loci have been identified (Engel et al. 2011). Other autoimmune disorders such as SLE have also been associated with increased rates of OCD (Bachen et al. 2009), and RA is associated with higher risk of concurrent mood and anxiety disorders (Nicassio 2010). Low but non-zero rates of SLE and type 1 diabetes were identified in the OCGAS cohort, both within the expected range. Higher-than-expected rates of RF—shared by first-degree relatives both with and without OCD—support the potential familial association suggested by preliminary studies. Further work that includes self-report data in healthy controls—and additional population-level epidemiological data—is needed to confirm and extend these findings. One limitation of existing national registries is the lack of detailed information on clinical phenotype, which may be relevant in understanding prognosis and response to conventional therapeutic agents in these individuals.

An association with frequent streptococcal infections has led to the hypothesis that some children with OCD have impaired humoral immune function (Swedo et al. 1998; Murphy et al. 2004). Indeed, a recent survey of 908 patients with PANS concluded that immune dysfunction is “pervasive,” with 53% of patients tested for some measure of immune status showing at least one abnormality, although there was no control group (Calaprice et al. 2017). In particular, 25% reported a diagnosis of immunodeficiency, most commonly hypogammaglobulinemia, although interpretation of these data is complicated by collection of samples during possible acute infection; moreover, based on typical assay protocols the actual prevalence of specific abnormalities (e.g., low immunoglobulin G) appeared comparable to expected values (Walker et al. 1994). There have also been preliminary reports of low IgA levels in patients with OCD compared with anxiety disorders or attention-deficit/hyperactivity disorder (Williams 2016).

While our data point to a higher-than-expected prevalence of meningitis or encephalitis (either infectious or autoimmune) as well as scarlet fever in both probands and non-OCD-affected first-degree relatives, further studies are needed to compare self-reported disease frequencies with a control group. Nevertheless, these data raise the possibility that sequelae of GAS infection are present at high rates and may be trait markers in families with a significant burden of OCD.

Moreover, our finding of increased cleaning obsessions and compulsions in patients reporting frequent ear or throat infections suggests that specific medical comorbidities—in this case, infections—may affect the content and severity of specific OCD symptoms. Alternatively, a patient's symptom severity may affect their perception of their medical history and reporting of illness. While there was no effect of comorbidity status on overall YBOCS score for the worst episode, other more sensitive differences in the course or expression of OCD—including episode duration, frequency, and mean severity—were not captured in this study.

Among immune-related comorbidities, we identified several associations consistent with previously reported epidemiological data (Fig. 1): RA with SLE and psoriasis (Lin et al. 1998; Alarcon-Segovia et al. 2005; Naldi and Mercuri 2010), MS and asthma with psoriasis (Fang et al. 2015; Egeberg et al. 2016), and RF with scarlet fever and throat/ear infections (Soderholm et al. 2018). We also found an association between IBD and RF, consistent with preliminary evidence for shared genetic risk (Abdallah et al. 2016) and data linking IBD with other immune-mediated diseases (Halling et al. 2017).

IBD includes Crohn's disease and ulcerative colitis, two chronic inflammatory disorders of the gastrointestinal tract. A recent systematic review described high rates of anxiety and depression in patients with IBD compared with healthy controls, particularly when the disease was active; children were also at increased risk of developing psychiatric comorbidities after IBD onset (Mikocka-Walus et al. 2016). While there may be etiological distinctions between childhood- and adult-onset IBD, the limited number of cases in the OCGAS cohort prevented any meaningful subgroup analysis. While our data do not support an association between IBD and OCD, larger population-based studies or multisite clinical registries are required given the low population frequency of individuals with both conditions.

It remains to be determined whether altered mucosal immunity involving the oropharynx—potentially leading to increased risk for scarlet fever and RF—might be causally related to the pathogenesis of OCD or simply represent nonspecific relationships among psychiatric and medical diseases. For example, the putative associations described herein may represent shared genetic and/or environmental risks predisposing individuals to both peripheral and central inflammation with neurodevelopmental consequences, including OCD (Hanamsagar and Bilbo 2017).

Alternatively, elements of both systemic immune disease and OCD may alter physiology so as to perpetuate symptoms of each other; proposed mechanisms involve the stress response, central activity of immunomodulatory signaling molecules such as histamine (Rapanelli et al. 2017), and alterations in microbiota (Sherwin et al. 2016; Rieder et al. 2017). The experience of both acute and chronic medical symptoms such as pain may also precipitate or modify OCD symptoms. There is also growing evidence that chronic psychiatric illnesses affect the expression and severity of immune-related diseases, both *in utero* (Douros et al. 2017; Van den Bergh et al. 2017) and later in life through altered glucocorticoid and beta-adrenergic activity (Ohno 2017).

While further studies facilitated by international multicenter collaborations are needed to better understand these relationships and underlying mechanisms, the true test of causality lies in interventional clinical trials. For example, a role for prostagalandin and thromboxane synthesis is suggested by efficacy of celecoxib as an adjuvant to fluvoxamine in OCD (Shalbafan et al. 2015), and other therapies that modulate mucosal immunity may also prove to be of benefit. Trials aimed at determining the effects of probiotic treatment in adults with OCD (NCT02334644) and of ibuprofen on functional magnetic resonance imaging activation patterns in the amygdala (NCT02507219) are currently ongoing.

Limitations of this study include potential bias associated with the retrospective nature of self-report in the OCGAS study (particularly for scarlet fever and RF, which require specific diagnostic criteria) and the absence of a control group. If present, however, the recollection bias appears to have affected probands and relatives for overlapping conditions (encephalitis or meningitis, scarlet fever, and RF), making it less likely that it is a random occurrence. Moreover, previous data suggest that the presence of an autoimmune disease based on self-report cannot be verified by checking medical records in up to 30% of cases (Broadley et al. 2000), and verification was not performed in this study. Similar error rates may be present in this study; although no data are available on recall bias specifically in patients with OCD, this population may overestimate personal risk of disease.

While we have attempted to thoroughly review available data and provide a broad estimate of population ranges, in some cases—particularly for nonreportable infectious diseases—these data are not available. Regional, temporal, and ethnic variations in prevalence among studies also limit the utility of calculated population rates in the context of a predominantly Caucasian population with a broad age range. In addition, the OCGAS medical questionnaire was limited to selected conditions and did not provide a comprehensive list of infectious, autoimmune, or inflammatory comorbidities. While conditions such as autoimmune thyroid diseases that are often difficult for patients to distinguish from nonautoimmune thyroid conditions without knowledge of laboratory testing were not included in this study, other categories such as encephalitis/meningitis were ambiguous with respect to etiology; although the vast majority of cases of meningitis are due to infection, approximately half of encephalitis cases may be autoimmune (Dubey et al. 2018).

Finally, given the relative rarity of autoimmune conditions in both the general population and in children with OCD, our data are limited by small sample sizes and low power to detect small differences between subgroups, including OCD-affected and nonaffected first-degree relatives. Nevertheless, our study is the first to carefully consider published population rates and is the largest descriptive study of immune-related comorbidities in childhood-onset OCD. These limitations, most present in previous smaller studies, highlight the need for further data in both general OCD and PANS/PANDAS populations that includes healthy controls together with means of verifying comorbidity diagnoses.

It is unclear whether our findings are specific to OCD or common among multiple psychiatric disorders. Other studies have provided circumstantial evidence for an association between immune-related disorders—including atopic disease and autoimmunity—and psychiatric comorbidities, including depression, schizophrenia, autism spectrum disorder, and other developmental disorders (Sweeten et al. 2003; Croen et al. 2005; Mouridsen et al. 2007; Gesundheit et al. 2013; Postal and Appenzeller 2015; Schans et al. 2017). Recent data suggest a link between stress-related disorders and subsequent autoimmune disease (Song et al. 2018). Moreover, other medical comorbidities, including migraines and respiratory diseases, are known to be more common among adults with OCD (Witthauer et al. 2014). Implementation of standardized medical comorbidity questionnaires across multiple pediatric psychiatric clinics may aid in determining whether particular comorbidities are associated with increased risk of specific psychiatric symptoms or diagnoses.

## Conclusions

Taken together, these data raise the possibility of an association between childhood-onset OCD and central nervous system infection or inflammation together with poststreptococcal autoimmunity. Delineation of clinical patterns of disease aggregation may provide mechanistic insight into this disease. Further multicenter controlled studies—in addition to larger population-based studies—are needed to characterize medical comorbidities in pediatric OCD and may aid in the development of therapeutic strategies targeting the immune response.

## Clinical Significance

Existing studies of immune-related comorbidities in childhood-onset OCD are limited by small sample sizes and restriction to assessment of PANS/PANDAS subtypes. This is the largest study to date describing immune-related comorbidities in a general population with childhood-onset OCD and points to high rates of certain inflammatory and infectious conditions in both affected individuals and their first-degree relatives. Our data are consistent with other recent studies suggesting that the increased prevalence of immune-related comorbidities may not be unique to PANS/PANDAS and provide an impetus for additional carefully designed research studies.

These findings also point to several considerations relevant to current clinical practice. A thorough medical history, including specific questions regarding chronic immune-related conditions as well as acute triggers such as infections should be considered in the diagnostic assessment of all children presenting with OCD symptoms. If identified, the impact of these medical comorbidities on OCD-related symptoms and functional status requires further assessment. Appropriate psychoeducation may include discussion of the multiple factors contributing to OCD risk, onset, and progression, including potential etiological links between the immune system and the brain that have also been described in other psychiatric disorders but about which our knowledge remains limited.

Clinical trials of adjunctive anti-inflammatory drugs, such as NSAIDS, may also be warranted in children with treatment-refractory OCD symptoms. Ultimately, further work is required to better understand the etiologic and prognostic implications of the associations identified in this study, with the goal of improving our understanding of OCD pathogenesis and facilitating the development of adjunctive immune-modulating therapeutic strategies.

## Supplementary Material

Supplemental data
