# Supplementary material for: Immune-Related Comorbidities in Childhood-Onset Obsessive Compulsive Disorder: Lifetime Prevalence in the Obsessive Compulsive Disorder Collaborative Genetics Association Study
Source: J Child Adolesc Psychopharmacol. 2019 Oct 7;29(8):615–24. doi: 10.1089/cap.2018.0140 (PMC6786333; doi:10.1089/cap.2018.0140)
Supplement: Supplemental data [file Supp_Data.pdf]

Supplementary Data

Supplementary Table S1. Previously Reported Prevalence of Immune-Mediated Comorbidities in Children with Obsessive Compulsive Disorder and Their First-Degree Relatives

References	*Diagnosis*	*Condition*	*Reported prevalence (%)*[a]			*Expected prevalence (%)*
			Child	*Mother*	*Father*	
Murphy et al. (2010) Clinical interview of 107 children with OCD and/or tic disorders.	OCD +/– tic disorder	Autoimmune disorders (total)	—	17.8[b]	—	4.7
		Hashimoto's thyroiditis	—	11.9	—	6.6
		Systemic lupus erythematosus	—	3.0[b]	—	0.09
		Rheumatic fever	—	3.0	—	1.49
		Rheumatoid arthritis	—	2.0	—	2.4
		Graves' disease	—	2.0	—	2.1
		Type 1 diabetes	—	0	—	2.8
		Guillain-Barré Syndrome	—	0	—	0.98
		Behcet disease	—	0	—	0.0019
		Crohn's disease	—	0	—	0.17
Stagi et al. (2014) Antibody screening in 77 consecutive patients fulfilling PANDAS criteria vs. 197 matched controls.	PANDAS	Overt hypothyroidism	1.2	—	—	0
		Subclinical hypothyroidism	3.8	—	—	3.6
		Autoimmune thyroiditis	2.46	—	—	1.14
		Celiac disease	1.2	—	—	0.05
		Autoantibodies	5.1	—	—	4.8
Yuce et al. (2014) Children and adolescents diagnosed with OCD only (*n*=26), TS only (*n*=32, OCD and TS (*n*=13), and healthy controls (*n*=35), evaluated by a pediatric allergist.	OCD + TS	Skin prick test positive	38.5[b]	—	—	5.7
		Any allergic disease	61.5[b]	—	—	22.9
		Asthma	23.1	—	—	8.6
		Allergic rhinitis	38.5	—	—	17.1
		Eczema	15.4[b]	—	—	5.7
		Family history of allergy	53.8	—	—	22.9
	OCD only	Skin prick test positive	21.9	—	—	5.7
		Any allergic disease	53.1[b]	—	—	22.9
		Asthma	18.8	—	—	8.6
		Allergic rhinitis	40.6[b]	—	—	17.1
		Eczema	9.4	—	—	5.7
		Family history of allergy	31.2	—	—	22.9
	TS	Skin prick test positive	26.9[b]	—	—	5.7
		Any allergic disease	61.5[b]	—	—	22.9
		Asthma	23.1	—	—	8.6
		Allergic rhinitis	38.5	—	—	17.1
		Eczema	26.9[b]	—	—	5.7
		Family hx of allergy	34.6	—	—	22.9

(*continued*)

Supplementary Table S1. (Continued)

References	*Diagnosis*	*Condition*	*Reported prevalence (%)*[a]			*Expected prevalence (%)*
			Child	*Mother*	*Father*	
Calaprice et al. (2017)	PANS	Autoimmune disorders (total)	—	20[b]	—	12.5
Clinical survey of 908 patients/ families with clinical diagnosis of PANS.		Frequent or chronic infection	73	10	3	—
		Allergies	55	43	30	—
		Frequent sore throats	51	—	—	—
		Frequent ear infections	44	—	—	—
		Eczema	37	17	11	—
		Pneumonia	32	—	—	—
		Asthma	27	16	11	—
		Scarlet fever	15	7	2	—
		Rheumatic fever	4	3	1	—
		Immune deficiency	—	4	1	—
		Lupus	—	2	0	—
		MS	—	1	0	—
		IBD	—	1	2	—
		Rheumatoid arthritis	—	4	1	—
		Hypothyroidism	7	16	4	—
		PANS	100	4	1	—
Frankovich et al. (2015)	OCD +/– PANS	Autoimmune or inflammatory disease	—	67 (all FDRs)		—
Chart review of 53 patients evaluated in PANS clinic representing a mix of PANS and classic OCD.		Inflammatory back pain	21	—		—
		At least one episode of arthritis	28	—		—
		Elevated ESR/CRP	7	—		—
		Presumed celiac disease	11	—		—
Dalsgaard et al. (2015) Prospective study of TS incidence based on national Danish health registers of 1.1 million children.	TS	100 autoimmune disorders (most common ulcerative colitis, rheumatoid arthritis, thyrotoxicosis, multiple sclerosis). Prevalence of SLE, pernicious anemia, celiac disease, and autoimmune thyroiditis was low (<10 cases).	Prior maternal autoimmune disease increased risk of TS in males (IRR 1.29, 1.05–1.58) but not females (IRR 0.89, 0.52–1.52)[c]. No single diagnosis was associated with TS.			

[a]Blank fields are shown where no data were included in the study.

[b]Difference from expected value was reported by authors as significant based on control group or epidemiological data.

[c]IRR with 95% confidence intervals are shown.

PANDAS, pediatric autoimmune neuropsychiatric disorder associated with streptococcal infections; PANS, pediatric acute-onset neuropsychiatric syndrome; TS, Tourette syndrome; FDR, first-degree relative; IRR, incidence risk ratio; OCD, obsessive compulsive disorder; SLE, systemic lupus erythematosus; IBD, inflammatory bowel disease; MS, multiple sclerosis; CRP, C-reactive protein; ESR, erythrocyte sedimentation rate.

Supplementary Table S2. Previously Published Estimates of Population Prevalence or Incidence of Immune-Mediated Diseases

Diagnosis	*Estimated lifetime prevalence (%)*	*Estimated incidence (% per year)*	*Location of study*	*Age group*	*Year(s) of data collection*	*Data summary*	*% Respondents endorsing ≥1 episode(s)*
Encephalitis or meningitis[a]	0.1–0.4	0.0072	United States of America	20–44	2000–2010	Between 2000 and 2010, there were 7.2 hospitalizations for encephalitis per 100,000 population, with higher rates in those <1 or >65 years (range 4.4–14.1 depending on age) (George et al. 2014). Incidence of meningococcal disease between ages 0 and 30 was estimated at 0.1–0.9 per 100,000 between 2006–2015, with total incidence in 2015 of 0.18 (Centers for Disease Control and Prevention 2017). Combining these figures for a rough estimate of incidence yields a maximum of 15 per 100,000 per year. A 2017 study identified a total of 26,429 patients with encephalitis or meningitis based on hospital discharge databases, an estimated 8.3 per 100,000 (Hasbun et al. 2017). These data likely represent underestimates given that not all cases require hospital admission.	1.4 (0.9–2.1)
Rheumatic fever[a]	0.1–0.2	3–20 cases per 100,000	United States of America	20–30	2000–2010	Lifetime prevalence at age 20–30 estimated based on CDC data and estimates based on North American epidemiological studies (Seckeler and Hoke 2011; Stockmann et al. 2012).	0.6 (0.3–1.2)
Scarlet fever[a]	1.0–2.0	0.01–0.06	Multiple		1983–2014	Combined studies. The 1983 Oxford surveillance program reported an incidence of 0.03% (Perks and Mayon-White 1983). Rates vary with region and time of year; associated with streptococcal infection. Recent data from Beijing (2005–2014) suggest an incidence of 14.25 per 100,000/year with 84% of cases among children 3–8 years of age (Mahara et al. 2016), from South Korea a rate of 13.7 per 100,000 (Park et al. 2017), and from Poland 59.5 with 96% of all cases in children under 15 (Staszewska-Jakubik et al. 2016).	4.0 (3.1–5.2)
Asthma	7.4–25.3	—	United States of America	25–34	2014–2015	Lifetime asthma prevalence based on 2015 CDC National Health Interview Survey. Estimate represents 95% confidence interval of the mean. Prevalence was lower in males (11.2%–12.9%) than females (13.3%–14.9%) (Centers for Disease Control and Prevention 2015a). There is significant regional variation (Centers for Disease Control and Prevention 2015b).	17.2 (15.0–19.8)

(*continued*)

Supplementary Table S2. (Continued)

Diagnosis	Estimated lifetime prevalence (%)	Estimated incidence (% per year)	Location of study	Age group	Year(s) of data collection	Data summary	% Respondents endorsing ≥1 episode(s)
Type 1 diabetes	0.2–0.6	—	United States of America	20–39	1999–2010	Estimate from the National Health and Nutrition Examination Survey based on age of diabetes diagnosis, the age of insulin initiation, and current use of insulin (Menke et al. 2013, 2015).	0.5 (0.2–1.3)
SLE	0.2–0.3	—	United States of America	>18	1988–2007	Combined studies. A 2015 study of a large United States Medicare population estimated prevalence at 0.3% (Garris et al. 2015). Older national prevalence data suggested a prevalence of 0.16%–0.32% with definite or probable SLE (Helmick et al. 2008).	0.3 (0.1–1.0)
Rheumatoid arthritis[a]	0.2–0.4	—	United States of America	18–34	2004–2014	A retrospective, cross-sectional study based on United States Administrative Health Insurance Claims Databases suggested an overall prevalence of 0.5% in 2014 (0.3% for males and 0.7%–0.8% for females) (Hunter et al. 2017). However, prevalence was lower among adults ages 18–34, ~0.2% in females and <0.1% in males, consistent with other United States studies (Crowson et al. 2011). Juvenile idiopathic arthritis (childhood-onset inflammatory arthritis) has a maximum prevalence of 0.1% (Harrold et al. 2013), which we have added to estimates for this age category.	1.0 (0.5–2.0)
Psoriasis	2.6–3.7	—	United States of America	20–59	2010–2013	Prevalence based on the National Health and Nutrition Examination Survey was estimated at 3.2% in 2010 (Rachakonda et al. 2014). A 95% confidence interval is shown. The prevalence among adults 20–29 years of age is likely lower (Helmick et al. 2014).	2.2 (1.4–3.4)
IBD	0.8–1.3	—	United States of America	25–44	2015	A 2015 study suggests self-reported rates based on data from the National Health Interview Survey are higher than those suggested by previous studies using administrative claims data (Dahlhamer et al. 2016). The 95% confidence interval from this study is shown.	1.1 (0.5–2.0)

Conditions with an estimated prevalence significantly greater than 0% are shown. Note that prevalence estimates based on the literature reflect cumulative lifetime prevalence but provide rough estimates only as they vary based on the study population, location, and age of participants. Where possible, a range based on available estimates is given. Specific lifetime prevalence data are provided for age ranges matching the average age at assessment of 27.8 years. Prevalence is estimated for cases in which only incidence data are available by multiplying by average age of study participants; this assumes only one episode per individual and similar rates over time (an incorrect assumption in some cases, e.g., for varicella zoster given differences in pre- and postvaccine rates).

[a]Rates identified in this study are greater than those identified in published literature.

IBD, inflammatory bowel disease; SLE, systemic lupus erythematosus; CDC, Centers for Disease Control and Prevention.

Supplementary Table S3. Obsessive Compulsive Disorder Symptom Severity Relative to Comorbidity

	Forbidden thoughts			*Symmetry*			*Cleaning*			*Hoarding*			*YBOCS*		
Medical comorbidity[a]	*Mean (SD)*	n	p[b]	*Mean (SD)*	n	p	*Mean (SD)*	n	P	*Mean (SD)*	n	p	*Mean (SD)*	n	p
Gender															
M	2.1 (1.1)	229	0.423	3.5 (1.5)	208	0.145	2.3 (1.0)	275	0.639	1.2 (0.9)	283	0.130	28.8 (6.5)	539	0.135
F	2.1 (1.0)	284		3.7 (1.5)	289		2.4 (1.0)	357		1.3 (0.9)	407		29.3 (6.2)	709	
Frequent ear or throat infections															
+	2.2 (1.0)	174	0.028	3.7 (1.5)	176	0.208	2.5 (0.9)	224	0.002*	1.4 (0.9)	241	0.016	28.9 (5.9)	445	0.444
−	2.0 (1.1)	338		3.5 (1.5)	320		2.3 (1.0)	404		1.2 (0.9)	444		29.2 (6.5)	788	
Scarlet fever															
+	2.4 (1.0)	18	0.217	3.6 (1.8)	16	0.893	2.5 (1.0)	21	0.584	1.4 (0.9)	25	0.438	28.9 (5.5)	46	0.876
−	2.1 (1.1)	489		3.6 (1.5)	475		2.4 (1.0)	602		1.3 (0.9)	660		29.1 (6.4)	1183	
Asthma[c]															
+	2.1 (1.0)	66	0.144	3.3 (1.6)	63	0.975	2.4 (0.9)	69	0.093	1.1 (1.0)	83	0.683	28.9 (6.6)	142	0.531
−	1.9 (1.1)	377		3.3 (1.5)	358		2.2 (1.0)	423		1.0 (0.9)	445		29.2 (6.3)	696	
Psoriasis															
+	1.9 (1.1)	12	0.905	3.6 (1.6)	12	0.557	2.3 (1.1)	10	0.725	1.1 (1.0)	14	0.679	30.9 (5.9)	19	0.211
−	2.0 (1.1)	431		3.3 (1.5)	409		2.2 (1.0)	481		1.0 (0.9)	512		29.2 (6.6)	818	
Infectious															
+	2.2 (1.0)	184	0.031	3.7 (1.5)	185	0.167	2.5 (0.9)	236	0.002*	1.4 (0.8)	257	0.016	28.8 (6.0)	475	0.311
−	2.0 (1.1)	329		3.5 (1.5)	312		2.3 (1.0)	396		1.2 (0.9)	433		29.1 (6.7)	773	
Autoimmune or inflammatory															
+	2.1 (1.0)	22	0.831	3.6 (1.4)	23	0.925	1.9 (1.2)	19	0.057	1.4 (0.9)	33	0.288	30.2 (6.2)	53	0.188
−	2.1 (1.0)	491		3.6 (1.5)	474		2.4 (1.0)	613		1.3 (0.9)	657		29.0 (6.3)	1195	
Any immune comorbidity															
+	2.2 (1.0)	222	0.033	3.6 (1.5)	220	0.392	2.5 (0.9)	272	0.020	1.3 (0.9)	307	0.025	28.9 (6.1)	558	0.378
−	2.0 (1.1)	291		3.5 (1.5)	277		2.3 (1.0)	360		1.2 (0.9)	383		29.2 (6.5)	690	

Factor structure of OCD symptoms assessed by the YBOCS Symptom Checklist. Symptom checklist and YBOCS score based on worst episode.

[a]Only conditions with at least 10 affected individuals completing the symptom checklist were included. All conditions were grouped into ‘‘infections,’’ ‘‘autoimmune or inflammatory,’’ and ‘‘any immune comorbidity’’ categories.

[b]*p*-value for continuous variables reflects two-sided Student’s *t*-test.

[c]There was a significant difference in age at OCD onset for patients with tic disorder (9.1 [3.2] vs. 10.2 [3.7] years, $n=1380$, $p<0.001$) and asthma (9.7 [3.2] vs. 10.6 [3.5] years, $n=946$, $p=0.001$), but not other comorbidities.

*$p<0.05$, remains significant with Bonferroni correction ($\alpha=0.05$, $n=8$).

YBOCS, Yale–Brown Obsessive Compulsive Scale; SD, standard deviation; OCD, obsessive compulsive disorder.

Supplementary Table S4. Associated Immune-Related Comorbidities Among Probands

Medical comorbidity (A)	*Associated comorbidity (B)*	*Cases (A)*	*Total (B)*	*Proportion (A/B) %*	*RR (CI)*[a]	n
Infectious						
Throat/ear infections	Scarlet fever	29	489	5.9	1.5 (1.2–2.0)	1371
	Rheumatic fever	5	490	1.0	2.3 (1.6–3.4)	1371
	Asthma	82	336	24.4	1.6 (1.3–1.9)	951
Scarlet fever	Throat/ear infections	29	55	52.7	2.0 (1.2–3.4)	1371
	Rheumatic fever	3	54	5.6	10.0 (3.9–25.4)	1367
Autoimmune						
Rheumatic fever	Throat/ear infections	5	6	83.3	9.0 (1.1–76.7)	1371
	Scarlet fever	3	8	37.5	14.6 (3.6–59.5)	1367
	IBD	1	3	33.3	46.7 (4.6–473.8)	943
SLE	Rheumatoid arthritis	1	3	33.3	46.8 (4.6–474.8)	945
Rheumatoid arthritis	SLE	1	10	10	34.9 (6.2–196.2)	945
	Psoriasis	2	10	20	11.6 (2.6–51.1)	946
Multiple sclerosis	Psoriasis	1	2	50	44.3 (2.9–684.4)	951
Inflammatory						
Psoriasis	Rheumatoid arthritis	2	20	10	10.4 (2.8–39.0)	946
	Multiple sclerosis	1	21	4.8	23.7 (5.6–101.4)	951
	Asthma	8	21	38.1	3.0 (1.3–7.1)	950
IBD	Rheumatic fever	1	10	10	34.8 (6.2–195.9)	943
Atopic						
Asthma	Throat/ear infections	82	163	50	1.9 (1.4–2.4)	951
	Psoriasis	8	162	4.9	2.3 (1.3–4.0)	950

Associations between immune-related comorbidities were determined by comparing the prevalence of comorbidity B among patients with comorbidity A compared with those without. Significant associations were identified by two-way comparisons using Chi-square tests with Bonferroni correction within each comorbidity. Corresponding relative risk is shown for significant associations ($n = 9/45$ pairwise comparisons).

[a]RR, relative risk of comorbidity B in patients with comorbidity A.

CI, 95% confidence interval; IBD, inflammatory bowel disease; SLE, systemic lupus erythematosus.

Supplementary References

Calaprice D, Tona J, Parker-Athill EC, Murphy TK: A survey of pediatric acute-onset neuropsychiatric syndrome characteristics and course. J Child Adolesc Psychopharmacol 27:607–618, 2017.

Centers for Disease Control and Prevention (CDC): 2015 Adult Asthma Data: Prevalence Tables and Maps. Behavioral Risk Factor Surveillance System National Center for Environmental Health. Available at: https://cdc.gov/asthma/brfss/2015/brfssdata.htm 2015a (last accessed November 27, 2017).

Centers for Disease Control and Prevention (CDC): 2015 National Health Interview Survey (NHIS) Data. National Center for Health Statistics. Available at: https://cdc.gov/nchs/nhis/index.htm 2015b (last accessed April 16, 2019).

Centers for Disease Control and Prevention (CDC): Meningococcal Disease: Technical and Clinical Information. National Center for Immunization and Respiratory Diseases. Available at: https://cdc.gov/meningococcal/clinical-info.html 2017 (last accessed March 28, 2017).

Crowson CS, Matteson EL, Myasoedova E, Michet CJ, Ernste FC, Warrington KJ, Davis JM, 3rd, Hunder GG, Therneau TM, Gabriel SE: The lifetime risk of adult-onset rheumatoid arthritis and other inflammatory autoimmune rheumatic diseases. Arthritis Rheum 63:633–639, 2011.

Dahlhamer J, Zammitti E, Ward B, Wheaton A, Croft J: Prevalence of inflammatory bowel disease among adults aged ≥18 years—United States, 2015. MMWR Morb Mortal Wkly Rep 65:1166–1169, 2016.

Dalsgaard S, Waltoft BL, Leckman JF, Mortensen PB: Maternal history of autoimmune disease and later development of tourette syndrome in offspring. J Am Acad Child Adolesc Psychiatry 54:495–501.e491, 2015.

Frankovich J, Thienemann M, Pearlstein J, Crable A, Brown K, Chang K: Multidisciplinary clinic dedicated to treating youth with pediatric acute-onset neuropsychiatric syndrome: Presenting characteristics of the first 47 consecutive patients. J Child Adolesc Psychopharmacol 25:38–47, 2015.

Garris C, Shah M, Farrelly E: The prevalence and burden of systemic lupus erythematosus in a medicare population: Retrospective analysis of medicare claims. Cost Eff Resour Alloc 13:9, 2015.

George BP, Schneider EB, Venkatesan A: Encephalitis hospitalization rates and inpatient mortality in the United States, 2000–2010. PLoS One 9:e104169, 2014.

Harrold LR, Salman C, Shoor S, Curtis JR, Asgari MM, Gelfand JM, Wu JJ, Herrinton LJ: Incidence and prevalence of juvenile idiopathic arthritis among children in a managed care population, 1996–2009. J Rheumatol 40:1218–1225, 2013.

Hasbun R, Rosenthal N, Balada-Llasat JM, Chung J, Duff S, Bozzette S, Zimmer L, Ginocchio CC: Epidemiology of meningitis and encephalitis in the United States, 2011–2014. Clin Infect Dis 65:359–363, 2017.

Helmick CG, Felson DT, Lawrence RC, Gabriel S, Hirsch R, Kwoh CK, Liang MH, Kremers HM, Mayes MD, Merkel PA, Pillemer SR, Reveille JD, Stone JH, National Arthritis Data Workgroup. Estimates of the prevalence of arthritis and other rheumatic conditions in the United States: Part I. Arthritis Rheum 58:15–25, 2008.

Helmick CG, Lee-Han H, Hirsch SC, Baird TL, Bartlett CL: Prevalence of psoriasis among adults in the U.S.: 2003–2006 and 2009–2010 National Health and Nutrition Examination Surveys. Am J Prev Med 47:37–45, 2014.

Hunter TM, Boytsov NN, Zhang X, Schroeder K, Michaud K and Araujo AB: Prevalence of rheumatoid arthritis in the United States

adult population in healthcare claims databases, 2004–2014. Rheumatol Int 37:1551–1557, 2017.

Mahara G, Wang C, Huo D, Xu Q, Huang F, Tao L, Guo J, Cao K, Long L, Chhetri JK, Gao Q, Wang W, Wang Q, Guo X: Spatiotemporal pattern analysis of scarlet fever incidence in Beijing, China, 2005–2014. Int J Environ Res Public Health 13:131, 2016.

Menke A, Casagrande S, Geiss L, Cowie CC: Prevalence of and trends in diabetes among adults in the United States, 1988–2012. JAMA 314:1021–1029, 2015.

Menke A, Orchard TJ, Imperatore G, Bullard KM, Mayer-Davis E, Cowie CC: The prevalence of type 1 diabetes in the United States. Epidemiology 24:773–774, 2013.

Murphy TK, Storch EA, Turner A, Reid JM, Tan J, Lewin AB: Maternal history of autoimmune disease in children presenting with tics and/or obsessive–compulsive disorder. J Neuroimmunol 229:243–247, 2010.

Park DW, Kim SH, Park JW, Kim MJ, Cho SJ, Park HJ, Jung SH, Seo MH, Lee YS, Kim BH, Min H, Lee SY, Ha DR, Kim ES, Hong Y, Chung JK: Incidence and characteristics of scarlet fever, South Korea, 2008–2015. Emerg Infect Dis 23:658–661, 2017.

Perks EM, Mayon-White RT: The incidence of scarlet fever. J Hyg (Lond) 91:203–209, 1983.

Rachakonda TD, Schupp CW, Armstrong AW: Psoriasis prevalence among adults in the United States. J Am Acad Dermatol 70:512–516, 2014.

Seckeler MD, Hoke TR: The worldwide epidemiology of acute rheumatic fever and rheumatic heart disease. Clin Epidemiol 3:67–84, 2011.

Stagi S, Rigante D, Lepri G, Bertini F, Matucci-Cerinic M, Falcini F: Evaluation of autoimmune phenomena in patients with pediatric autoimmune neuropsychiatric disorders associated with streptococcal infections (PANDAS). Autoimmun Rev 13:1236–1240, 2014.

Staszewska-Jakubik E, Czarkowski MP, Kondej B: Scarlet fever in Poland in 2014. Przegl Epidemiol 70:195–202, 2016.

Stockmann C, Ampofo K, Hersh AL, Blaschke AJ, Kendall BA, Korgenski K, Daly J, Hill HR, Byington CL, Pavia AT: Evolving epidemiologic characteristics of invasive group a streptococcal disease in Utah, 2002–2010. Clin Infect Dis 55:479–487, 2012.

Yuce M, Guner SN, Karabekiroglu K, Baykal S, Kilic M, Sancak R, Karabekiroglu A: Association of Tourette syndrome and obsessive-compulsive disorder with allergic diseases in children and adolescents: A preliminary study. Eur Rev Med Pharmacol Sci 18: 303–310, 2014.
